# Perceived learning outcomes partly mediate the association between interactive features and adolescent satisfaction in documentary-based digital learning

**DOI:** 10.3389/fpsyg.2026.1906522

**Published:** 2026-09-10

**Authors:** Yue Zhang, Mohd Nor Shahizan Ali, Mohd Azul Bin Mohamad Salleh, Miao Xu

**Affiliations:** 1 Faculty of Social Sciences and Humanities, National University of Malaysia (UKM), Bangi, Selangor, Malaysia; 2 College of Art and Creativity, Anhui University of Applied Technology, Hefei, Anhui, China

**Keywords:** adolescent media use, documentary-based digital learning, perceived interactivity, perceived learning outcomes, social cognitive theory, structural equation modeling, user satisfaction, uses and gratifications theory

## Abstract

**Introduction:**

Digital platforms increasingly present adolescents with documentary-based educational content that combines audiovisual narratives with navigation, feedback, personalization, and social interaction. However, the association between perceived interactive features and post-use satisfaction, including the possible mediating role of perceived learning outcomes, remains unclear. Guided by Uses and Gratifications Theory and Social Cognitive Theory, this study proposed an Interactive Affordance-Cognition-Outcome framework and examined whether perceived learning outcomes statistically mediate the association between interactive features and satisfaction.

**Methods:**

A cross-sectional questionnaire was completed by 562 adolescents aged 13-18 recruited from selected eastern, central, and western regions of China.

**Results:**

Structural equation modeling indicated that interactive features were positively associated with perceived learning outcomes (*β* = 0.534, *p* < 0.001); perceived learning outcomes were positively associated with satisfaction (*β* = 0.649, *p* < 0.001); and the direct association between interactive features and satisfaction remained significant (*β* = 0.448, *p* < 0.001). Bootstrap analysis with 5,000 resamples showed a significant indirect effect of 0.347 [SE = 0.028; bias-corrected 95% CI = (0.293,0.404)], indicating complementary partial mediation.

**Discussion:**

The findings indicate that perceived interactive features are associated with satisfaction both directly and through adolescents’ perceptions of knowledge acquisition, competence, self-efficacy, and cognitive growth. By examining the indirect association through perceived learning outcomes, the study connects communication-oriented gratification with social-cognitive learning processes and offers design implications for digital platforms, educators, and families.

## 1 Introduction

Digital communication technologies have changed how adolescents obtain information, encounter cultural narratives, and organize self-directed learning. The 5th National Survey Report on Minors’ Internet Use reported that China had 193 million minor internet users by the end of 2022 and that internet penetration among minors had reached 97.2% (Department of Rights Protection for Adolescents of the Central Committee of the Communist Youth League of China and China Internet Network Information Center, 2023). Short-video services, knowledge communities, and platform-based learning resources therefore form an important part of adolescents’ everyday information environment. Documentary and historical educational content has become increasingly visible within this environment. On platforms such as Bilibili, Douyin, Kuaishou, Xiaohongshu, and Zhihu, documentary materials may be accompanied by nonlinear navigation, commenting, recommendation systems, interactive maps, timelines, quizzes, or immersive extensions. These features allow users to select, reorganize, discuss, and revisit content rather than only receive a fixed audiovisual sequence. Immersive documentary research also suggests that the mode of presentation can shape emotional engagement and attitudes, highlighting the importance of examining how technological affordances influence user experience (Gallego Abellán et al., 2024). Research on adolescent digital engagement similarly shows that platform use is embedded in broader social, cultural, and educational conditions (Liu, 2025).

The present study uses the term documentary-based digital learning to refer to documentary, historical, and explanatory audiovisual content encountered through digital platforms and evaluated by adolescents as a learning resource. The focus is not on a single production format or platform. Instead, the study examines perceived interactive features across four dimensions: responsiveness, user control, personalization, and connectivity. This definition reduces the conceptual ambiguity that would arise from treating all short videos as interactive documentaries or from assuming that a particular documentary title is inherently interactive. Prior studies of online learning have established that course design, self-efficacy, self-regulation, and perceived value are associated with learning satisfaction (Al-Fraihat et al., 2020; Zheng and Xiao, 2024). However, less is known about the cognitive pathway associated with the relationship between platform interactivity and satisfaction in documentary-based adolescent learning. Interactivity may create immediate enjoyment and a sense of agency, but it may also help users recognize gains in knowledge, skills, confidence, and cognitive understanding. Treating these two pathways as conceptually distinct is important because a platform can be enjoyable without being educationally meaningful, while perceived learning can strengthen satisfaction beyond momentary entertainment.

Uses and Gratifications Theory views audiences as active users who select media to satisfy cognitive, affective, and social needs (Katz et al., 1973). Social Cognitive Theory explains how environmental affordances, self-efficacy, cognitive appraisal, and expected outcomes jointly shape behaviour and experience (Bandura, 1986). Based on the above, this study proposes an Interactive Affordance-Cognition-Outcome framework, where interactive features serve as perceived media affordances, perceived learning outcomes function as a candidate cognitive pathway, and adolescent satisfaction constitutes the post-use outcome. Therefore, the three questions of this study are: whether perceived interactive features are associated with perceived learning outcomes; whether perceived learning outcomes are related to adolescent satisfaction; and whether an indirect statistical association between interactive features and satisfaction exists through perceived learning outcomes. The first is the idea. First, this study presents a clear-cut model of documentary-based digital learning and does not consider platform use as a single, undifferentiated behaviour. Second, it links communication-oriented gratification with a social-cognitive learning path. Third, it separates the direct experience path from the indirect cognitive path and tests both in the same structural equation model.

The main contributions are summarized as follows:

(1) This study builds an Interactive Affordance–Cognition–Outcome framework to integrate Uses and Gratifications Theory with Social Cognitive Theory and explore how perceived interactive features relate to adolescent satisfaction.
(2) Perceived learning outcomes are proposed as a specific candidate cognitive path linking interactive features to satisfaction. The model divides the indirect learning-related path from the direct experience path and shows that both occur simultaneously in a complementary partial mediation structure.
(3) The study operationalizes interactive features through responsiveness, user control, personalization, and connectivity, thereby providing a more clearly bounded and multidimensional understanding of interactivity in documentary-based digital learning.
(3) The study extends digital-learning research beyond formal online courses by examining voluntary and entertainment-oriented documentary, historical, and explanatory media environments used by adolescents.

(4) The rest of this paper is organised as follows. Section 2 reviews the literature on interactive functions, perceived learning results, adolescent satisfaction, and the theoretical basis for the proposed framework. Section 3 introduces the research model and develops the hypotheses. Section 4 is the research design, measurement tools, sample selection process, data collection and analysis methods. Section 5 presents the measurement-model assessment, structural-model results, hypothesis testing and mediation analysis. Section 6 is the research results, theoretical contributions, practical values, deficiencies and prospects for future research. Section 7 is the Conclusion.

## 2 Literature review

### 2.1 Interactive features in documentary-based digital learning

Interactivity can be a technology feature, a way of interacting, or a person’s subjective sense. Satisfaction and Learning research are concerned with Perceived Interactivity, and the same technical function may be experienced differently by different users. Building on perceived-interactivity research (McMillan and Hwang, 2002), this study shows interactive features in four ways. The swiftness of a response after being exposed to a stimulus. Control is the user’s selection of the sequence, repeated parts, speed adjustment and other route modifications according to the content. Personalisation refers to how recommendations and content organisation consider users’ interests or previous behaviour. A network will provide a place for people to communicate, share, and learn online.

In documentary-based learning, the above attributes can be implemented as branching navigation, interactive timelines, searchable transcripts, quizzes, annotations, recommendation systems and social discussions. These attributes may reduce passive viewing by requiring selection and interpretation. They can also offer multiple exposures, comparisons of different views, and additional explanations. However, more interactivity is not always better. Unaligned Functions may result in inattentiveness or superficial exploration. Therefore, the problem is not whether a platform has interactive functions, but whether young people believe that these functions provide a sense of purpose.

### 2.2 Perceived learning outcomes

Perceived learning outcomes are the learners’ evaluations of what they believe they have gained from an experience. The construct is not objectively verifiable behaviour; rather, it refers to learners’ sense of learning, skill growth, self-efficacy and cognitive development. Although the perceived results do not always match the actual outcomes, they are necessary for the post-use assessment because learners respond to what they believe a particular activity has helped them learn or achieve.

Social Cognitive Theory is applicable; that is, interactive experiences can enhance self-efficacy through repeated successful behaviour, feedback, and observation of other users, and recognition of progress (Bandura, 1986). In online learning, self-efficacy and self-regulated learning are closely linked to course satisfaction and engagement (Zheng and Xiao, 2024; Li et al., 2023). Perceived learning will thus be used as a near-term cognitive evaluation system to determine the educational value of interactive affordances.

### 2.3 Adolescent satisfaction

Adolescent satisfaction refers to their overall evaluation of the documentary-based learning content and platform environment after using it. The construct includes satisfaction with the content, the platform and the learning experience, as well as continuance and word-of-mouth intentions as conative expressions of a favourable evaluation. The above high-order operationalization is based on the idea that satisfaction includes not only immediate approval but also a desire to continue participating and recommending the service. Studies of e-learning satisfaction have also stressed system quality, interaction and perceived value, and intention to continue using a learning environment (Al-Fraihat et al., 2020; Leong et al., 2021; Jiang et al., 2021).

Satisfaction for adolescent users will be provided by two or more reasons. First, there is the experience of pleasure: control, novelty, responsiveness and social participation can all be enjoyable. The second is cognitive gratification; that is, the platform can help users learn about a certain subject, organise information, and feel more competent. Therefore, a model that includes both direct and indirect paths can be used to explain why interactivity is still associated with satisfaction even after considering perceived learning outcomes.

### 2.4 Interactive affordance-cognition-outcome framework

The three links of the proposed framework are as follows: The interactive-affordance stage is the sense of responsiveness, control, personalization, and connectivity in adolescents. Cognitive-appraisal stage: Perception of learning outcomes in knowledge, skills, self-efficacy and cognitive enhancement. The Post-Use Outcome Stage Records Adolescent Satisfaction. Uses and Gratifications Theory explains why people use media to meet their needs for information, experience and socialisation; Social Cognitive Theory explains how interaction and feedback are converted into beliefs about one’s own competence and learning.

The two paths are as follows. The indirect pathway proposes an association between interactive features and satisfaction through perceived learning outcomes. The direct path is a form of gratification that does not require full-fledged learning and includes enjoyment, agency, novelty, and socialisation. If both paths are still significant, the model suggests complementary partial mediation rather than full mediation (Zhao et al., 2010).

## 3 Research model and hypotheses

### 3.1 Research model

**Figure 1:**
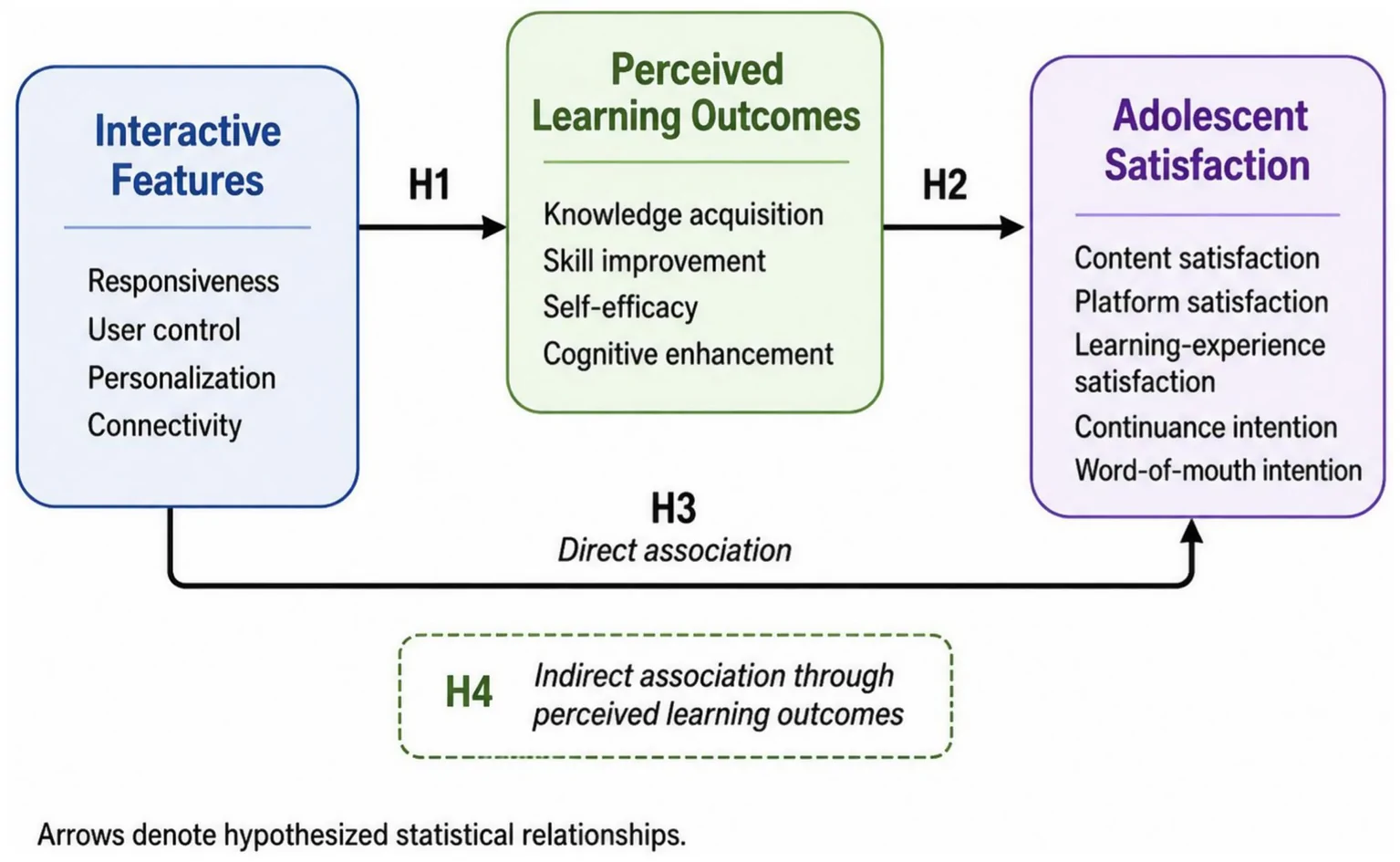
Conceptual research model and hypothesized mediation paths.

Figure 1 presents the three-construct research model. Interactive Features are taken as the focus predictor, Perceived Learning Outcomes serve as the mediator, and Adolescent Satisfaction is the post-use outcome. The diagram shows the three direct model paths (H1–H3) and the indirect association tested in H4; the arrows represent hypothesized statistical relationships rather than experimentally established causal effects.

### 3.2 Research hypotheses

Interactive features may be associated with greater active engagement by providing timely feedback, choice, adaptive content, and opportunities for exchange. These affordances may help adolescents revisit difficult information, connect new content to prior interests, and observe the consequences of their choices. Such experiences are expected to be positively associated with adolescents’ perceptions of growth in knowledge, skills, confidence, and understanding. Therefore:

*H1*: Interactive features are positively associated with perceived learning outcomes.

Learners who report stronger perceived learning outcomes are also expected to report greater satisfaction. When adolescents believe that an experience has supported useful knowledge or competence gains, they may evaluate that experience more favorably. This expectation is consistent with evidence linking self-efficacy, perceived value, and learning progress with satisfaction in digital learning environments (Al-Fraihat et al., 2020; Zheng and Xiao, 2024). Therefore:

*H2*: Perceived learning outcomes are positively associated with adolescent satisfaction.

Interactive features may also be associated with satisfaction independently of perceived learning. Responsiveness can reduce frustration, control can strengthen agency, personalization can increase relevance, and connectivity can provide social gratification. These immediate experiential benefits justify testing a direct statistical association between interactivity and satisfaction. Therefore:

*H3*: Interactive features are directly and positively associated with adolescent satisfaction.

H1 and H2 jointly imply an indirect statistical pathway in which interactive features are associated with satisfaction through perceived learning outcomes. If the indirect effect is significant while the direct effect remains significant in the same direction, the observed pattern is consistent with complementary partial mediation (Zhao et al., 2010). Therefore:

*H4*: Perceived learning outcomes statistically mediate the association between interactive features and adolescent satisfaction.

## 4 Methodology

### 4.1 Research design

This study adopted a quantitative cross-sectional survey design to examine associations among perceived interactive features, perceived learning outcomes, and adolescent satisfaction in documentary-based digital learning. Structural equation modeling (SEM) was used to evaluate the proposed framework, including a hierarchical measurement model with three higher-order constructs and 13 first-order dimensions, the direct structural paths, and the indirect association between interactive features and adolescent satisfaction through perceived learning outcomes.

The target population comprised adolescents aged 13–18 who had experience viewing documentary, historical, or explanatory audiovisual content through commonly used digital platforms. Data were collected from September to December 2024 in 12 cooperating schools across eastern, central, and western China, with four schools in each macro-region. The main-sample allocation was 205 respondents from eastern China, 184 from central China, and 173 from western China. Schools were selected on the basis of accessibility within each macro-region, after which participating classes were randomly selected within cooperating schools. School identities were omitted to protect the site. Eligible students were aged 13–18, had previous experience with the focal type of digital content, and had completed the relevant consent or assent procedure. As the sample was not taken from an all-encompassing national probability-sampling frame, it is regarded as relatively diverse in regions rather than nationally representative.

The cross-sectional design permits examination of theoretically specified associations and an indirect effect at one point in time, but it cannot establish temporal precedence or causality. Structural coefficients are therefore interpreted as model-based associations rather than evidence of experimental effects. Questionnaires were administered in classroom settings using standardized instructions, and trained research personnel explained the study purpose and completion procedures without directing participants toward particular responses. The achieved sample size reflects the class-based field recruitment (620 questionnaires distributed; 562 usable responses).

No *a priori* power calculation was performed for the field recruitment. For context, the achieved *N* = 562 provides about 80% power at a two-sided α = 0.05 level to detect a bivariate association of around *r* = 0.118. Sensitivity estimates indicate the obtained sample and are not the results of *a priori* SEM power analysis.

### 4.2 Measurement instruments

The 39 Likert-type measurement items of the three focal constructs and the separate demographic section made up the questionnaire. The 39 indicators of measurement are grouped into 13 first-level dimensions and three higher-level constructs. All scale items are five-point response items, ranging from 1 (strongly disagree) to 5 (strongly agree). All scale items were on a five-point scale, and the extremes were “strongly disagree” (1) and “strongly agree” (5). Three experts in digital communication and educational psychology reviewed the instrument for content relevance, age-appropriate language and clarity. A pilot survey with 68 adolescents was used to refine ambiguous wording and confirm that the instructions and five-point response format were understandable. In the pilot sample, Cronbach’s alpha was 0.893 for Interactive Features, 0.844 for Perceived Learning Outcomes, and 0.884 for Adolescent Satisfaction; the corresponding scale means were 3.168, 2.960, and 2.994, with composite-score standard deviations of 0.779, 0.706, and 0.686. Supplementary material S1 provides the complete 39-item questionnaire, item codes, 13-dimension mapping, response anchors, source and adaptation notes, English and Chinese wording, and pilot-test documentation. The questionnaire was developed in Chinese for the target population; Supplementary material S1 includes the English wording for cross-language reference. No formal forward-backward translation procedure was applied.

**Figure 2:**
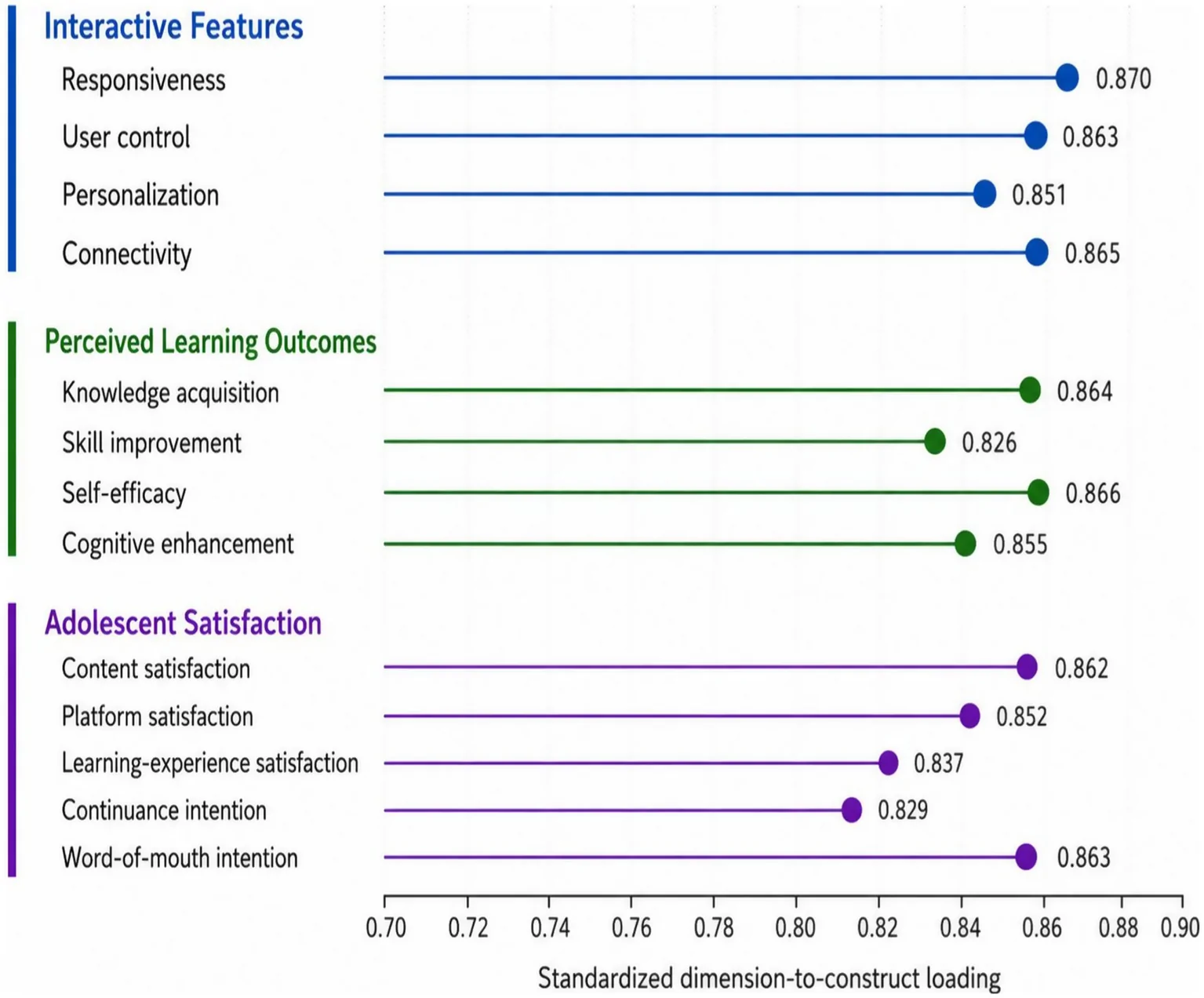
Complete dimension-to-construct loadings for the hierarchical measurement model.

Interactive features were operationalized with 12 items representing responsiveness, user control, personalization, and connectivity, with three items per dimension. McMillan and Hwang’s perceived-interactivity framework provides the principal conceptual anchor for perceived responsiveness and user control, and the wording was contextualized for contemporary documentary-based platform use (McMillan and Hwang, 2002). Perceived Learning Outcomes comprised 12 items covering knowledge acquisition, skill improvement, self-efficacy, and cognitive enhancement; this construct was theoretically grounded in Social Cognitive Theory and informed by contemporary online-learning research (Zheng and Xiao, 2024; Bandura, 1986). Adolescent Satisfaction comprised 15 items covering content satisfaction, platform satisfaction, learning-experience satisfaction, continuance intention, and word-of-mouth intention; the construct wording was informed by e-learning satisfaction and continued-use research (Al-Fraihat et al., 2020; Leong et al., 2021). The hierarchical measurement specification therefore comprised 39 items organized into 13 first-order dimensions and three higher-order constructs. Figure 2 reports the 13 dimension-to-construct loadings, whereas the 39 item-level loadings are reported in Supplementary material S1.

### 4.3 Sampling and data collection

Data were collected from September to December 2024 from students in Grades 7–12 across eastern, central, and western China. A total of 620 questionnaires were distributed, and 562 were validly collected; thus, the actual collection rate reached 90.6%. The 12 cooperating schools provided 205 respondents from eastern China, 184 from central China and 173 from western China. Middle-school students were in grades 7–9, and high-school students were in grades 10–12. Preferred platform was recorded as a single-response demographic item. Exposure was observational; the subjects were not given a single documentary title, a fixed viewing time, or an identical platform interface, but rather experienced documentaries, history or explanation-oriented audiovisual materials in their daily lives. Table 1 shows the general information.

**Table 1:** Demographic characteristics of respondents (*N* = 562).

Variable	Category	Frequency (*N*)	Percentage (%)
Gender	Male	278	49.5
	Female	284	50.5
Age	13 years	88	15.7
	14 years	113	20.1
	15 years	149	26.5
	16 years	105	18.7
	17–18 years	107	19.0
Grade	Middle school	297	52.8
	High school	265	47.2
Platform	Bilibili	177	31.5
	Douyin	132	23.5
	Kuaishou	113	20.1
	Xiaohongshu	86	15.3
	Zhihu	54	9.6

**Figure 3:**
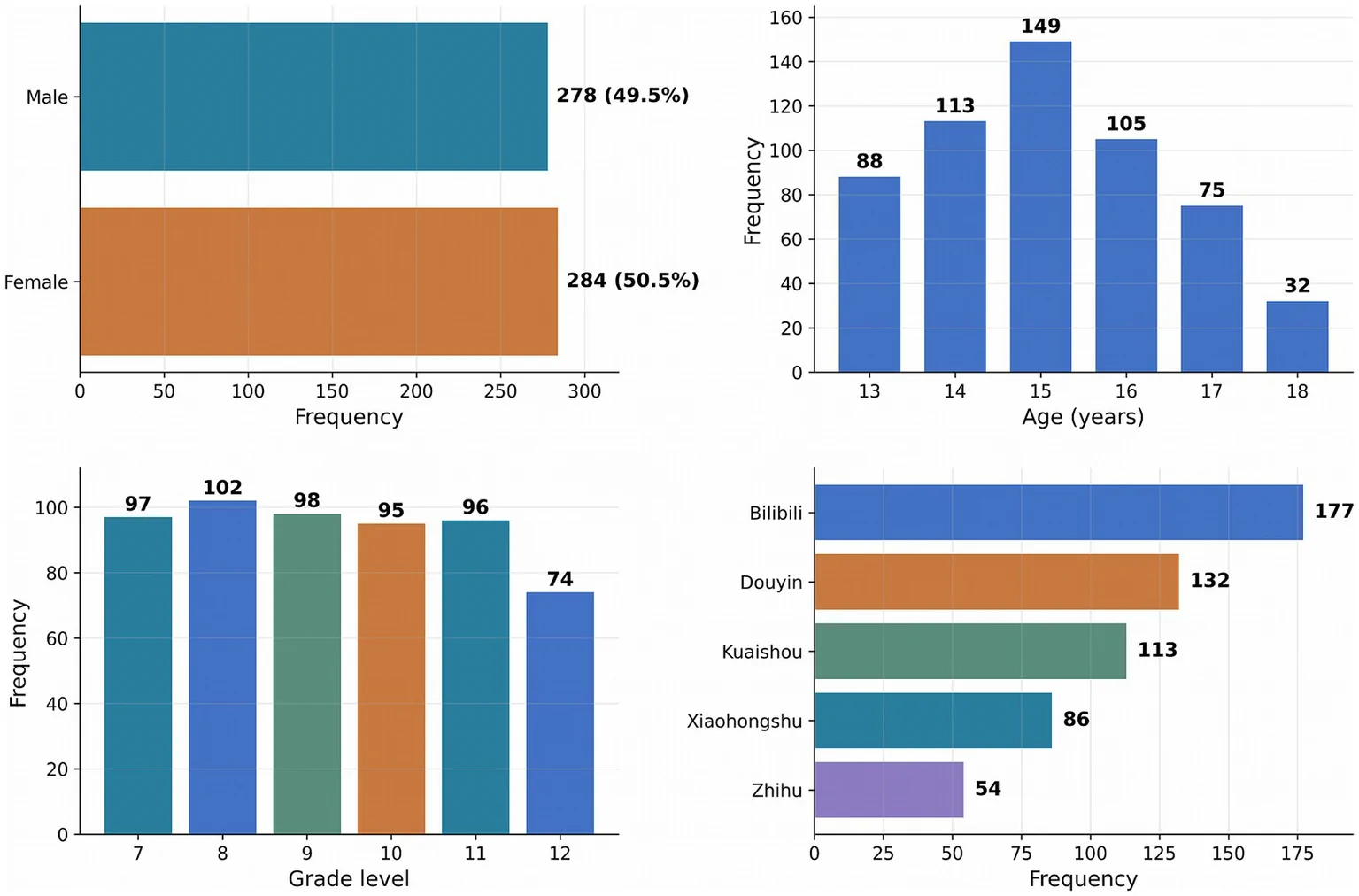
Sample demographic distributions across gender, age, grade, and preferred platform.

The sample was nearly gender balanced. Based on the disaggregated age frequencies, the mean age was 15.11 years (SD = 1.43). Middle-school students represented 52.8% of the sample and high-school students represented 47.2%. Bilibili was the most frequently selected platform, followed by Douyin, Kuaishou, Xiaohongshu, and Zhihu. Table 1 uses the aggregated age and school-level categories, whereas Figure 3 provides the corresponding disaggregated age and grade distributions.

### 4.4 Data analysis

SPSS 26.0 was used for data screening, descriptive statistics, reliability analysis, correlations, and Harman’s single-factor test. AMOS 24.0 was used for confirmatory factor analysis (CFA) and structural equation modeling. Model fit was evaluated using the chi-square to degrees-of-freedom ratio, comparative fit index, Tucker-Lewis index, root mean square error of approximation, and standardized root mean square residual. Following commonly used recommendations, an acceptable fit was indicated by a chi-square to degrees-of-freedom ratio below 3, CFI and TLI above 0.90, and RMSEA and SRMR below 0.08 (Hu and Bentler, 1999).

Internal consistency was evaluated using Cronbach’s alpha and composite reliability. Convergent validity was examined using standardized loadings and average variance extracted (AVE). Discriminant validity was assessed with the Fornell-Larcker criterion (Fornell and Larcker, 1981) and the heterotrait-monotrait ratio (HTMT), using 0.85 as a conservative screening threshold (Henseler et al., 2015). Item-level CFA diagnostics were evaluated for all 39 items under the 39-item → 13-dimension → 3-construct hierarchy. Standardized item loadings, residual variances, higher-order factor correlations, and residual diagnostics are reported in Supplementary material S1. Common method bias was assessed using Harman’s single-factor test and a latent common-method-factor comparison, consistent with the recommendation that multiple diagnostic procedures be used rather than relying on a single test (Podsakoff et al., 2003). Latent-variable SEM estimates constitute the primary inferential results, whereas composite-score correlations and construct-score regressions are used for descriptive and robustness analyses.

The indirect effect was evaluated using 5,000 bootstrap resamples and a bias-corrected 95% confidence interval. An indirect effect was considered statistically significant when the interval did not contain zero (Hayes, 2013). Table 2 reports zero-order correlations from composite scores, whereas Section 5.4 reports standardized latent-variable estimates obtained simultaneously in the SEM. The two coefficient sets reflect distinct analytic levels and may therefore differ in magnitude.

**Table 2:** Means, standard deviations, and inter-construct correlations.

Construct	Mean	SD	1	2	3
1. Interactive features	3.15	0.74	(0.862)		
2. Perceived learning outcomes	2.95	0.73	0.600^**^	(0.853)	
3. Adolescent satisfaction	2.96	0.70	0.489^**^	0.685^**^	(0.849)

Diagonal values in parentheses are square roots of AVE. ^**^p < 0.01.

## 5 Results

### 5.1 Common method bias

Because all focal variables were collected through adolescent self-report in the same questionnaire and at a single time point, common method variance was examined before testing the structural model. First, all measurement items were entered into an unrotated exploratory factor analysis. Eight factors had eigenvalues greater than 1, and the first factor accounted for 34.72% of the total variance, below the 40% screening threshold adopted in the study. This result indicates that no single general factor dominated the item covariance.

Second, a latent common-method-factor specification was compared with the baseline hierarchical measurement model. Adding the method factor changed both CFI and RMSEA by less than 0.01. Taken together, the two diagnostics did not indicate a dominant common-method factor. Response-style and social-desirability effects nevertheless remain possible because all focal constructs were measured by self-report at one time point.

### 5.2 Measurement model

The hierarchical measurement model with three higher-order constructs and 13 first-order dimensions showed acceptable fit: χ2/df=2.847 , CFI=0.953 , TLI=0.941 , RMSEA=0.057 , and SRMR=0.042 . All 13 reported dimension-to-construct loadings exceeded 0.70 and were statistically significant ( p<0.001 ). Figure 2 presents the complete set of 13 dimension-level loadings; Table 3 reports the AVE and CR values for the three constructs.

**Table 3:** Reliability and convergent-validity assessment.

Construct	Cronbach’s alpha	CR	AVE	Square root of AVE
Interactive features	0.884	0.921	0.743	0.862
Perceived learning outcomes	0.874	0.914	0.727	0.853
Adolescent satisfaction	0.903	0.928	0.720	0.849

As reported in Table 3, all three constructs demonstrated satisfactory reliability and convergent validity. Interactive Features showed a Cronbach’s alpha of 0.884, a composite reliability of 0.921, and an AVE of 0.743. Perceived Learning Outcomes yielded corresponding values of 0.874, 0.914, and 0.727, while Adolescent Satisfaction recorded the highest internal consistency, with a Cronbach’s alpha of 0.903 and a composite reliability of 0.928, together with an AVE of 0.720. All composite reliability values exceeded the recommended threshold of 0.70, and all AVE values were above 0.50. In addition, the standardized loadings ranged from 0.826 to 0.870. As a result, the observed scales have good internal consistency and convergent validity.

Item-level CFA diagnostics showed standardized item-to-dimension loadings from 0.637 to 0.778 and standardized residual variances from 0.395 to 0.594. The supplementary hierarchical CFA yielded higher-order latent-factor correlations of 0.731 for Interactive Features–Perceived Learning Outcomes, 0.571 for Interactive Features–Adolescent Satisfaction, and 0.813 for Perceived Learning Outcomes–Adolescent Satisfaction. The CFA specification included no cross-loadings or correlated residuals, and the largest absolute off-diagonal standardized residual was 0.087. Supplementary Tables S1, S2 report the complete item-level loading/residual table and factor-correlation matrix.

### 5.3 Discriminant validity and descriptive relationships

Fornell-Larcker Criterion and HTMT were employed to test discriminant validity. The diagonal square roots of AVE in Table 2 exceed the corresponding zero-order composite-score correlations. As shown in Table 4, the HTMT ratios for Interactive Features–Perceived Learning Outcomes, Interactive Features–Adolescent Satisfaction, and Perceived Learning Outcomes–Adolescent Satisfaction were 0.684, 0.542 and 0.772, respectively. All three values were less than the conservative threshold of 0.85, providing supporting evidence for discriminant validity.

**Table 4:** Heterotrait-Monotrait ratios from the item-level dataset.

Construct pair	HTMT	Threshold	Assessment
Interactive features–perceived learning outcomes	0.684	<0.85	Pass
Interactive features–adolescent satisfaction	0.542	<0.85	Pass
Perceived learning outcomes–adolescent satisfaction	0.772	<0.85	Pass

The mean for interactive features was slightly above the scale midpoint of 3.00. The means for perceived learning outcomes and adolescent satisfaction were slightly below the midpoint, indicating generally moderate rather than uniformly high evaluations.

### 5.4 Structural model and hypothesis testing

**Figure 4:**
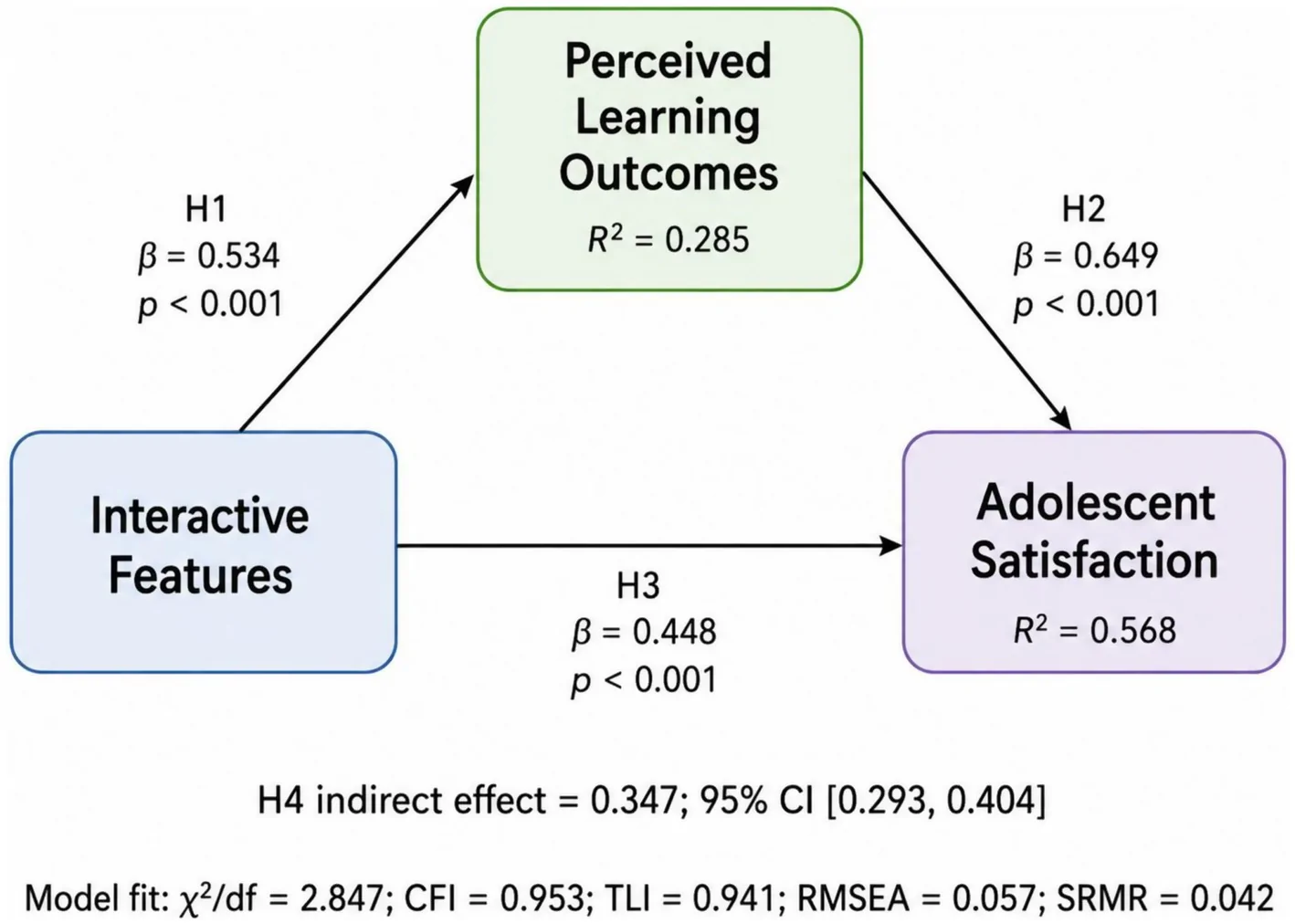
Simplified structural-model path estimates and explained variance.

As illustrated in Figure 4, the structural model retained an acceptable fit: χ^2^/df = 2.847, CFI = 0.953, TLI = 0.941, RMSEA = 0.057, and SRMR = 0.042. Because all theoretically specified directional paths among the three latent constructs were estimated, the global fit values were equivalent to those of the corresponding measurement model. Figure 4 summarizes the path estimates and explained variance; Table 5 provides the detailed hypothesis statistics.

**Table 5:** Summary of hypothesis testing.

Hypothesis	Relationship/effect	Estimate	SE	*t* or 95%CI	*p*	Result
H1	IF → PLO	0.534	0.048	11.125	<0.001	Supported
H2	PLO → Satisfaction	0.649	0.051	12.725	<0.001	Supported
H3	IF → Satisfaction	0.448	0.052	8.615	<0.001	Supported
H4	IF → PLO → Satisfaction	0.347	0.028	[0.293, 0.404]	<0.001	Supported

H1–H3 are standardized latent-variable path estimates from the AMOS SEM; H4 is the bootstrap indirect effect based on 5,000 resamples.

H1 was supported: interactive features were positively associated with perceived learning outcomes (β = 0.534, SE = 0.048, *t* = 11.125, *p* < 0.001). Interactive features explained R2=0.285 of the variance in perceived learning outcomes. H2 was supported: perceived learning outcomes were positively associated with adolescent satisfaction (β = 0.649, SE = 0.051, *t* = 12.725, *p* < 0.001). H3 was also supported: interactive features retained a positive direct association with adolescent satisfaction after perceived learning outcomes were included (β = 0.448, SE = 0.052, *t* = 8.615, *p* < 0.001). Together, the predictors accounted for R2=0.568 of the variance in adolescent satisfaction.

### 5.5 Mediation effect

Bootstrap analysis with 5,000 resamples yielded an indirect effect estimate of 0.347 (SE = 0.028). The bias-corrected 95% CI = [0.293, 0.404] did not include zero, supporting H4. The direct IF-to-satisfaction path also remained positive and statistically significant after the mediator was included. Following Zhao et al. (2010), this pattern is statistically consistent with complementary partial mediation: interactive features are associated with satisfaction both through perceived learning outcomes and through an additional direct association. Given the cross-sectional design, the indirect effect represents a statistical association; temporal causation requires longitudinal or experimental evidence.

### 5.6 Robustness and alternative specification checks

A supplementary construct-score model including preferred platform was used to assess the robustness of the focal associations alongside the primary latent-variable SEM. With Bilibili as the reference category, adding platform indicators did not materially alter the focal associations: the Interactive Features–Perceived Learning Outcomes association remained positive (*β* = 0.601, *p* < 0.001), and in the satisfaction model the associations for Interactive Features (*β* = 0.116, *p* = 0.003) and Perceived Learning Outcomes (*β* = 0.616, *p* < 0.001) remained significant. The platform interaction block was not significant for the Perceived Learning Outcomes model (Wald *χ*^2^(4) = 6.97, *p* = 0.137), and the combined Interactive Features × platform and Perceived Learning Outcomes × platform interactions were not significant in the satisfaction model (Wald *χ*^2^(8) = 5.68, *p* = 0.683). Therefore, the focal construct-score associations were not affected by the preferred-platform control in this sample. Analysis of coefficient robustness; Measurement invariance across platforms requires separate formal tests.

A second sensitivity analysis separated the nine direct satisfaction items (SAT1-SAT9) from continuance intention (SAT10-SAT12) and word-of-mouth intention (SAT13-SAT15). In standardized regressions controlling for Interactive Features and Perceived Learning Outcomes, core satisfaction strongly predicted continuance intention (*β* = 0.594, *p* < 0.001, *R*^2^ = 0.466) and word-of-mouth intention (*β* = 0.536, *p* < 0.001, *R*^2^ = 0.469). This alternative specification supports the interpretation of continuance and word-of-mouth as downstream behavioural intentions. The main model takes Adolescent Satisfaction as the general post-use indicator, and the sensitivity model considers continuance and word-of-mouth as the subsequent intentions. Supplementary Tables S4, S5 present the full coefficients.

## 6 Discussion

### 6.1 Interpretation of the findings

The results support the proposed Interactive Affordance-Cognition-Outcome framework. Interactive Functions were positively correlated with perceived learning outcomes. Responsiveness, user control, personalisation and connectivity are all in line with the idea that they can help young people learn to use documentary-based platforms effectively. In line with Social Cognitive Theory, this pattern also includes feedback, successful behaviour, observation and a sense of control over one’s own life and results in terms of self-efficacy. Adolescents will be more willing to report learning achievements in their daily lives if they can use the platform independently, receive personalised feedback, connect learning materials with their interests, and participate in discussions or sharing.

Perceived learning outcomes were most closely associated with adolescent satisfaction in the structural model. Based on the above results, satisfaction with documentary-based digital learning is associated with students’ sense of learning gains in knowledge and skills, self-efficacy and understanding, as well as entertainment or convenience. This is in line with other studies on online learning that have linked self-efficacy, perceived value and learning progress to satisfaction and continuous use (Al-Fraihat et al., 2020; Zheng and Xiao, 2024).

The direct relationship between interactive features and satisfaction remained significant after perceived learning outcomes were included. This result supports the Uses and Gratifications perspective that users may derive immediate benefits from agency, relevance, rapid feedback, and social participation. These benefits may be associated with a more favorable experience even when the user does not explicitly interpret them as learning gains. The direct and indirect paths therefore represent different but complementary statistical patterns rather than competing explanations.

The significant bootstrap indirect effect is statistically consistent with partial mediation. An indirect association was found; therefore, it can be concluded that interactivity and satisfaction are related through adolescents’ self-assessments of their learning. At the same time, the remaining direct path indicates that perceived learning outcomes do not account for the full interactivity-satisfaction association. Together, the direct and indirect effects support a dual-path interpretation of the interactivity-satisfaction association.

### 6.2 Theoretical contributions

Combining Uses and Gratifications Theory with Social Cognitive Theory, this study presents a parsimonious three-stage model to extend existing research. Uses and Gratifications Theory explains the value that users derive from interactive media affordances, whereas Social Cognitive Theory provides a basis for interpreting the association between these affordances and cognitive appraisals of learning and competence. Their integration extends the conceptualization of interactivity beyond a technical platform attribute and supports interpretation of how interactive functions may acquire psychological and educational value for adolescent users.

A further contribution lies in examining perceived learning outcomes as a distinct cognitive appraisal associated with the relationship between interactive features and satisfaction. Most previous research has treated interactivity, engagement, perceived usefulness and satisfaction as closely related predictors without differentiating between experiential gratification and cognitively based evaluation. The proposed model distinguishes these pathways by showing that interactive features are associated with satisfaction both directly, through agency, relevance, responsiveness, and social participation, and indirectly, through adolescents’ perceptions of knowledge acquisition, skill development, self-efficacy, and cognitive growth. The two paths together offer a more specific explanation for the complementary partial mediation in digital learning environments.

This study also extends digital-learning research to documentary-based learning on popular social and video platforms. Platform-mediated documentary viewing is often voluntary, fragmented, personalized, and embedded in entertainment-oriented environments. The observed learning-related association suggests that meaningful learning appraisals can also be reported outside structured instructional settings. Therefore, the new system will extend the application of communication and social cognition to informal, audiovisual and platform-mediated learning.

### 6.3 Limitations and future research

Several methodological and interpretive limitations should be considered when evaluating the findings. The cross-sectional design cannot establish temporal order or causality, and reverse or reciprocal relations remain plausible. For example, adolescents who are already more satisfied or engaged may retrospectively rate platform interactivity and their learning gains more favorably. Longitudinal studies should test temporal ordering, experimental studies should manipulate specific features, and competing structural models should compare plausible reverse-path specifications.

The reliance on self-reported perceived learning outcomes also limits the interpretation of learning-related findings. Perceived learning outcomes were assessed through self-report and may not correspond directly to objective knowledge retention, transfer, or critical understanding. Future research should combine subjective evaluations with quizzes, delayed-recall tests, source-evaluation tasks, and behavioral trace data. Common method variance also remains a design-level concern despite the Harman and latent-method-factor diagnostics because all focal constructs were measured from the same respondents at one time point.

Sampling and clustered recruitment structures impose additional restrictions on the generalizability and statistical inference. The sample was geographically diverse but not acquired by national probability sampling. Respondents were nested within classes and schools, but the reported SEM treated observations at the individual level. If there is significant within-school or within-class dependence, the standard errors may be understated. Future research can keep the cluster structure from the analysis, report intraclass correlations and design effects, and use cluster-robust or multilevel SEM if necessary. The above sampling and clustering features limit the generalisation of the recruitment network.

Heterogeneity in content exposure and platform characteristics represents another important boundary condition. Exposure was heterogeneous by design. The study grouped documentary, historical, and explanatory content within a broad category of platform-mediated educational media and did not standardize one title, viewing duration, or interface across respondents. Platform heterogeneity remains an important boundary condition because Bilibili (31.5%), Douyin (23.5%), Kuaishou (20.1%), Xiaohongshu (15.3%), and Zhihu (9.6%) differ in video length, recommendation logic, navigation, commenting, and opportunities for sustained engagement (Montag et al., 2021). The supplementary platform check did not detect a significant interaction block for the focal composite-score relationships. Because preferred platform was represented by a single self-reported category, formal measurement invariance remains untested. Future platform-specific work should standardize exposure more tightly and use measurement-invariance testing and multi-group or multilevel SEM.

Adolescent Satisfaction was operationalized in the primary model as a broad post-use evaluation containing content, platform, and learning-experience satisfaction together with continuance and word-of-mouth intentions. The alternative analysis showed that the nine core satisfaction items strongly predicted both continuance and word-of-mouth intentions when these were treated as downstream outcomes. These results support the broad post-use interpretation of the primary construct. Future confirmatory studies should prespecify and compare the broad higher-order and downstream-intention specifications.

Further measurement validation would strengthen the generalizability of the present measurement structure. The item-level CFA and HTMT diagnostics reported here improve measurement transparency, but replication with independent samples and formal cross-language equivalence testing remains desirable (Henseler et al., 2015; Brislin, 1970).

## 7 Conclusion

This study examined how perceived interactive features relate to adolescent satisfaction in documentary-based digital learning. The three-construct model showed positive associations between interactive features and perceived learning outcomes, between perceived learning outcomes and satisfaction, and between interactive features and satisfaction. Bootstrap analysis identified a significant indirect statistical association through perceived learning outcomes while the direct association remained significant, a pattern consistent with complementary partial mediation. The findings therefore support two coexisting model-based pathways: an experiential pathway associated with agency, relevance, responsiveness, and social participation, and a learning-related pathway associated with perceived gains in knowledge, skills, confidence, and understanding. Effective documentary-based learning design should connect interactive functions to clear cognitive purposes and make progress visible without reducing educational value to engagement metrics. Because the evidence is cross-sectional, self-reported, platform-heterogeneous, and potentially clustered within schools and classes, causal conclusions require longitudinal, experimental, multilevel, and objective-performance evidence.

## Data Availability

The datasets presented in this study can be found in online repositories. The names of the repository/repositories and accession number(s) can be found in the article/Supplementary material.
