# Supplementary material for: Perceived learning outcomes partly mediate the association between interactive features and adolescent satisfaction in documentary-based digital learning
Source: Front Psychol. 2026 Sep 10;17:1906522. doi: 10.3389/fpsyg.2026.1906522 (PMC13601317; doi:10.3389/fpsyg.2026.1906522)
Supplement: Supplementary file 1 [file Table_1.docx]

**Supplementary Material S1**

**Participant Questionnaire Used in the Study**

**39-Item Questionnaire on Interactive Features, Perceived Learning Outcomes, and Adolescent Satisfaction**

Associated manuscript: "Perceived learning outcomes partly mediate the association between interactive features and adolescent satisfaction in documentary-based digital learning"

**Instructions / 作答说明**

本问卷面向13-18岁、具有通过数字平台观看纪录片、历史类或解释性视听内容经验的青少年。请根据您平时使用此类内容时的真实体验作答。

This questionnaire is for adolescents aged 13-18 who have experience viewing documentary, historical, or explanatory audiovisual content through digital platforms. Please answer according to your typical experience.

量表说明 / Response scale: 1 = 非常不同意 / Strongly disagree; 2 = 不同意 / Disagree; 3 = 既不同意也不反对 / Neither agree nor disagree; 4 = 同意 / Agree; 5 = 非常同意 / Strongly agree.

每题请选择一个选项 / Select one response for each statement.

**Part A. Background Information / A部分：基本信息**

A1. 性别 / Gender: ( ) 男 / Male	( ) 女 / Female

A2. 年龄 / Age: ( ) 13	( ) 14	( ) 15	( ) 16	( ) 17	( ) 18

A3. 学段 / School level: ( ) 初中 / Middle school	( ) 高中 / High school

A4. 主要使用平台 / Preferred platform: ( ) Bilibili	( ) Douyin	( ) Kuaishou	( ) Xiaohongshu	( ) Zhihu

**Part B. Interactive Features (12 items) / B部分：互动特征（12题）**

**Responsiveness / 响应性**

1. (IF1) 平台能够快速响应我的选择或操作。
*The platform responds quickly to my choices or inputs.*
( ) 1	( ) 2	( ) 3	( ) 4	( ) 5

2. (IF2) 当我与内容互动时，我能够及时获得反馈或回应。
*When I interact with the content, I receive feedback or responses without unnecessary delay.*
( ) 1	( ) 2	( ) 3	( ) 4	( ) 5

3. (IF3) 平台提供的回应或反馈清晰，并与我当前的操作相关。
*The responses or feedback provided by the platform are clear and relevant to what I am doing.*
( ) 1	( ) 2	( ) 3	( ) 4	( ) 5

**User Control / 用户控制**

4. (IF4) 我可以轻松控制观看内容的顺序或节奏。
*I can easily control the order or pace in which I view the content.*
( ) 1	( ) 2	( ) 3	( ) 4	( ) 5

5. (IF5) 我可以根据学习需要暂停、重播、返回或跳过某些部分。
*I can pause, replay, revisit, or skip sections according to my learning needs.*
( ) 1	( ) 2	( ) 3	( ) 4	( ) 5

6. (IF6) 我可以选择下一步要查看的内容部分或相关信息。
*I can choose which part of the content or related information to explore next.*
( ) 1	( ) 2	( ) 3	( ) 4	( ) 5

**Personalization / 个性化**

7. (IF7) 平台会推荐符合我兴趣的纪录片或解释性内容。
*The platform recommends documentary or explanatory content that matches my interests.*
( ) 1	( ) 2	( ) 3	( ) 4	( ) 5

8. (IF8) 平台向我呈现的内容或相关材料会反映我之前的观看选择或偏好。
*The content or related materials presented to me reflect my previous viewing choices or preferences.*
( ) 1	( ) 2	( ) 3	( ) 4	( ) 5

9. (IF9) 平台使学习体验与我的个人兴趣或需要相关。
*The platform makes the learning experience feel relevant to my individual interests or needs.*
( ) 1	( ) 2	( ) 3	( ) 4	( ) 5

**Connectivity / 连接性**

10. (IF10) 平台为我提供与其他用户评论或讨论内容的机会。
*The platform gives me opportunities to comment on or discuss the content with other users.*
( ) 1	( ) 2	( ) 3	( ) 4	( ) 5

11. (IF11) 在观看或学习过程中，我可以与其他用户分享观点、问题或有用信息。
*I can share viewpoints, questions, or useful information with other users while engaging with the content.*
( ) 1	( ) 2	( ) 3	( ) 4	( ) 5

12. (IF12) 与其他用户互动有助于我联系与内容有关的不同观点或解释。
*Interaction with other users helps me connect different ideas or interpretations related to the content.*
( ) 1	( ) 2	( ) 3	( ) 4	( ) 5

**Part C. Perceived Learning Outcomes (12 items) / C部分：感知学习成果（12题）**

**Knowledge Acquisition / 知识获得**

13. (PLO1) 观看这些内容有助于我更好地理解相关主题。
*Viewing this content helps me understand the topic better.*
( ) 1	( ) 2	( ) 3	( ) 4	( ) 5

14. (PLO2) 我能从这些内容中学到新的事实、概念或背景知识。
*I learn new facts, concepts, or background information from this content.*
( ) 1	( ) 2	( ) 3	( ) 4	( ) 5

15. (PLO3) 观看后，我觉得自己获得了以前不了解的有用知识。
*After viewing the content, I feel that I have gained useful knowledge that I did not have before.*
( ) 1	( ) 2	( ) 3	( ) 4	( ) 5

**Skill Improvement / 技能提升**

16. (PLO4) 这种学习体验提高了我查找和整理相关信息的能力。
*This learning experience improves my ability to find and organize relevant information.*
( ) 1	( ) 2	( ) 3	( ) 4	( ) 5

17. (PLO5) 这种学习体验提高了我比较、解释或总结某一主题信息的能力。
*This learning experience improves my ability to compare, explain, or summarize information about a topic.*
( ) 1	( ) 2	( ) 3	( ) 4	( ) 5

18. (PLO6) 使用此类内容后，我觉得自己更能理解和解读纪录片、历史或解释性信息。
*I feel better able to interpret documentary, historical, or explanatory information after using this type of content.*
( ) 1	( ) 2	( ) 3	( ) 4	( ) 5

**Self-efficacy / 自我效能**

19. (PLO7) 这种体验使我对自主学习更有信心。
*This experience makes me feel more confident about learning independently.*
( ) 1	( ) 2	( ) 3	( ) 4	( ) 5

20. (PLO8) 接触这些内容后，我觉得自己能够独立理解类似主题。
*I feel capable of understanding similar topics on my own after engaging with this content.*
( ) 1	( ) 2	( ) 3	( ) 4	( ) 5

21. (PLO9) 我有信心有效使用数字平台的功能来支持自己的学习。
*I feel confident that I can use digital-platform features effectively to support my learning.*
( ) 1	( ) 2	( ) 3	( ) 4	( ) 5

**Cognitive Enhancement / 认知提升**

22. (PLO10) 这些内容有助于我把新观点与已有知识联系起来。
*This content helps me connect new ideas with what I already know.*
( ) 1	( ) 2	( ) 3	( ) 4	( ) 5

23. (PLO11) 这种学习体验促使我更深入地思考原因、关系或后果。
*This learning experience encourages me to think more deeply about causes, relationships, or consequences.*
( ) 1	( ) 2	( ) 3	( ) 4	( ) 5

24. (PLO12) 这些内容有助于我从多个角度思考一个主题。
*This content helps me consider a topic from more than one perspective.*
( ) 1	( ) 2	( ) 3	( ) 4	( ) 5

**Part D. Adolescent Satisfaction (15 items) / D部分：青少年满意度（15题）**

**Content Satisfaction / 内容满意度**

25. (SAT1) 我对所观看纪录片或解释性内容的教育价值感到满意。
*I am satisfied with the educational value of the documentary or explanatory content I view.*
( ) 1	( ) 2	( ) 3	( ) 4	( ) 5

26. (SAT2) 这些内容总体上符合我学习该主题的预期。
*The content generally meets my expectations for learning about the topic.*
( ) 1	( ) 2	( ) 3	( ) 4	( ) 5

27. (SAT3) 总体而言，我认为观看此类内容是有价值的。
*Overall, I consider this type of content worthwhile to watch.*
( ) 1	( ) 2	( ) 3	( ) 4	( ) 5

**Platform Satisfaction / 平台满意度**

28. (SAT4) 我对平台支持我的观看和学习体验的方式感到满意。
*I am satisfied with how the platform supports my viewing and learning experience.*
( ) 1	( ) 2	( ) 3	( ) 4	( ) 5

29. (SAT5) 平台的功能使我使用此类内容的整体体验令人满意。
*The platform functions make my experience with this type of content satisfactory.*
( ) 1	( ) 2	( ) 3	( ) 4	( ) 5

30. (SAT6) 总体而言，当我使用基于纪录片的学习内容时，平台环境能够满足我的需要。
*Overall, the platform environment meets my needs when I use documentary-based learning content.*
( ) 1	( ) 2	( ) 3	( ) 4	( ) 5

**Learning-Experience Satisfaction / 学习体验满意度**

31. (SAT7) 我对自己从此类数字内容中学到的东西感到满意。
*I am satisfied with what I learn from this type of digital content.*
( ) 1	( ) 2	( ) 3	( ) 4	( ) 5

32. (SAT8) 整体学习体验对我而言既愉快又有收获。
*The overall learning experience is enjoyable and rewarding for me.*
( ) 1	( ) 2	( ) 3	( ) 4	( ) 5

33. (SAT9) 总体而言，我对通过数字平台上的纪录片、历史或解释性内容进行学习的体验感到满意。
*Overall, I am satisfied with my experience of learning through documentary, historical, or explanatory content on digital platforms.*
( ) 1	( ) 2	( ) 3	( ) 4	( ) 5

**Continuance Intention / 持续使用意愿**

34. (SAT10) 我打算继续使用数字平台观看纪录片、历史或解释性内容进行学习。
*I intend to continue using digital platforms to view documentary, historical, or explanatory content for learning.*
( ) 1	( ) 2	( ) 3	( ) 4	( ) 5

35. (SAT11) 今后我很可能继续选择这些平台上的类似学习型内容。
*I am likely to choose similar learning-oriented content on these platforms in the future.*
( ) 1	( ) 2	( ) 3	( ) 4	( ) 5

36. (SAT12) 我愿意再次花时间使用此类平台内容进行学习。
*I would be willing to spend time using this type of platform content for learning again.*
( ) 1	( ) 2	( ) 3	( ) 4	( ) 5

**Word-of-Mouth Intention / 口碑推荐意愿**

37. (SAT13) 我愿意向朋友或同学推荐此类基于纪录片的数字学习内容。
*I would recommend this type of documentary-based digital learning content to friends or classmates.*
( ) 1	( ) 2	( ) 3	( ) 4	( ) 5

38. (SAT14) 我愿意向他人介绍自己通过此类内容获得的有益学习体验。
*I would tell other people about useful learning experiences I have had with this type of content.*
( ) 1	( ) 2	( ) 3	( ) 4	( ) 5

39. (SAT15) 当其他青少年想了解某个主题时，我会鼓励他们使用类似的数字平台内容。
*I would encourage other adolescents to use similar digital-platform content when they want to learn about a topic.*
( ) 1	( ) 2	( ) 3	( ) 4	( ) 5

**问卷结束 / End of Questionnaire**

**感谢您的参与**。 / Thank you for completing the questionnaire.

**Methodological Documentation Accompanying the Questionnaire**

The following methodological information documents the questionnaire structure, source basis, language development, pilot testing, and supplementary analyses reported with the manuscript. It is not part of the respondent-facing questionnaire.

**1. Instrument Structure**

The 39 measurement items comprise Interactive Features (12 items), Perceived Learning Outcomes (12 items), and Adolescent Satisfaction (15 items). They are organized into 13 first-order dimensions: four for Interactive Features, four for Perceived Learning Outcomes, and five for Adolescent Satisfaction. These dimensions represent three higher-order constructs in the SEM reported in the manuscript.

**2. Source Basis and Adaptation Status**

Interactive Features. McMillan and Hwang (2002) served as the conceptual anchor for perceived interactivity, especially responsiveness/time and user control. The four-dimension structure and 12 item wordings were contextualized for digital documentary platforms.

Perceived Learning Outcomes. Bandura (1986) and Zheng and Xiao (2024) provided theoretical grounding for self-efficacy, cognitive appraisal, and perceived learning in online environments. The 12 items are context-specific operationalizations informed by these sources.

Adolescent Satisfaction. Al-Fraihat et al. (2020) and Leong et al. (2021) provided conceptual support for e-learning satisfaction, perceived value, continued use, and post-use evaluation. The 15 items operationalize content, platform, and learning-experience satisfaction together with continuance and word-of-mouth intentions.

**3. Language and Instrument Development**

The questionnaire was developed in Chinese for adolescent participants. Item wording was adapted for clarity, comprehensibility, and suitability for the target population. The English wording in this supplement is provided for reporting and cross-language reference. Formal forward-backward translation was not used.

Three specialists in digital communication and educational psychology reviewed the questionnaire for content relevance, clarity, and age-appropriate wording. Their feedback informed wording refinements before pilot testing.

**4. Pilot-Test Record**

A pilot survey with 68 adolescents was conducted before the main survey to refine ambiguous wording and confirm that the instructions and five-point response format were understandable. In the pilot sample, Cronbach's alpha was 0.893 for Interactive Features, 0.844 for Perceived Learning Outcomes, and 0.884 for Adolescent Satisfaction. The corresponding scale means were 3.168, 2.960, and 2.994, with composite-score standard deviations of 0.779, 0.706, and 0.686. Pilot feedback informed the wording of the final 39-item instrument across 13 dimensions.

**5. Documented Dimension-to-Construct Loadings**

Interactive Features - Responsiveness (IF1-IF3): 0.870

Interactive Features - User Control (IF4-IF6): 0.863

Interactive Features - Personalization (IF7-IF9): 0.851

Interactive Features - Connectivity (IF10-IF12): 0.865

Perceived Learning Outcomes - Knowledge Acquisition (PLO1-PLO3): 0.864

Perceived Learning Outcomes - Skill Improvement (PLO4-PLO6): 0.826

Perceived Learning Outcomes - Self-efficacy (PLO7-PLO9): 0.866

Perceived Learning Outcomes - Cognitive Enhancement (PLO10-PLO12): 0.855

Adolescent Satisfaction - Content Satisfaction (SAT1-SAT3): 0.862

Adolescent Satisfaction - Platform Satisfaction (SAT4-SAT6): 0.852

Adolescent Satisfaction - Learning-Experience Satisfaction (SAT7-SAT9): 0.837

Adolescent Satisfaction - Continuance Intention (SAT10-SAT12): 0.829

Adolescent Satisfaction - Word-of-Mouth Intention (SAT13-SAT15): 0.863

These are the 13 dimension-level coefficients reported in Figure 3 of the manuscript and are distinct from the 39 item-to-dimension loadings. Section 6 reports the complete item-level loadings, residual variances, latent-factor correlations, and residual diagnostics.

**6. Complete Item-Level CFA Diagnostics**

Item-level CFA diagnostics were evaluated for the 562 × 39 main-sample item matrix under the 39-item → 13-dimension → 3-construct hierarchy. The manuscript's latent-variable SEM remains the primary inferential model. This supplementary hierarchical CFA documents the item-level loading layer, standardized residual variances, higher-order latent-factor correlations, and residual diagnostics.

**Table S1. Standardized item-to-dimension CFA loadings and residual variances.**

| **Item** | **First-order dimension** | **Std. loading** | **Residual variance** |
| --- | --- | --- | --- |
| IF1 | Responsiveness | 0.686 | 0.530 |
| IF2 | Responsiveness | 0.690 | 0.524 |
| IF3 | Responsiveness | 0.736 | 0.458 |
| IF4 | User Control | 0.751 | 0.436 |
| IF5 | User Control | 0.737 | 0.457 |
| IF6 | User Control | 0.710 | 0.496 |
| IF7 | Personalization | 0.778 | 0.395 |
| IF8 | Personalization | 0.683 | 0.534 |
| IF9 | Personalization | 0.669 | 0.553 |
| IF10 | Connectivity | 0.725 | 0.475 |
| IF11 | Connectivity | 0.737 | 0.456 |
| IF12 | Connectivity | 0.668 | 0.553 |
| PLO1 | Knowledge Acquisition | 0.667 | 0.556 |
| PLO2 | Knowledge Acquisition | 0.686 | 0.529 |
| PLO3 | Knowledge Acquisition | 0.707 | 0.500 |
| PLO4 | Skill Improvement | 0.645 | 0.584 |
| PLO5 | Skill Improvement | 0.697 | 0.514 |
| PLO6 | Skill Improvement | 0.690 | 0.523 |
| PLO7 | Self-efficacy | 0.688 | 0.527 |
| PLO8 | Self-efficacy | 0.672 | 0.548 |
| PLO9 | Self-efficacy | 0.656 | 0.570 |
| PLO10 | Cognitive Enhancement | 0.696 | 0.516 |
| PLO11 | Cognitive Enhancement | 0.696 | 0.516 |
| PLO12 | Cognitive Enhancement | 0.637 | 0.594 |
| SAT1 | Content Satisfaction | 0.695 | 0.518 |
| SAT2 | Content Satisfaction | 0.728 | 0.470 |
| SAT3 | Content Satisfaction | 0.700 | 0.510 |
| SAT4 | Platform Satisfaction | 0.722 | 0.479 |
| SAT5 | Platform Satisfaction | 0.695 | 0.516 |
| SAT6 | Platform Satisfaction | 0.706 | 0.501 |
| SAT7 | Learning-Experience Satisfaction | 0.704 | 0.504 |
| SAT8 | Learning-Experience Satisfaction | 0.669 | 0.553 |
| SAT9 | Learning-Experience Satisfaction | 0.707 | 0.500 |
| SAT10 | Continuance Intention | 0.705 | 0.504 |
| SAT11 | Continuance Intention | 0.719 | 0.482 |
| SAT12 | Continuance Intention | 0.705 | 0.503 |
| SAT13 | Word-of-Mouth Intention | 0.692 | 0.521 |
| SAT14 | Word-of-Mouth Intention | 0.732 | 0.465 |
| SAT15 | Word-of-Mouth Intention | 0.694 | 0.519 |

All 39 standardized item loadings were positive and ranged from 0.637 to 0.778. Standardized residual variances ranged from 0.395 to 0.594. The CFA specification included no cross-loadings or correlated residuals. The largest absolute off-diagonal standardized residual was 0.087.

**Table S2. Higher-order latent-factor correlations from the supplementary hierarchical CFA.**

| **Factor 1** | **Factor 2** | **Latent correlation** |
| --- | --- | --- |
| Interactive Features | Perceived Learning Outcomes | 0.731 |
| Interactive Features | Adolescent Satisfaction | 0.571 |
| Perceived Learning Outcomes | Adolescent Satisfaction | 0.813 |

**7. HTMT Discriminant-Validity Analysis**

HTMT was calculated from the item-level correlation matrix using 0.85 as the conservative screening threshold (Henseler et al., 2015). The ratios were 0.684 for Interactive Features–Perceived Learning Outcomes, 0.542 for Interactive Features–Adolescent Satisfaction, and 0.772 for Perceived Learning Outcomes–Adolescent Satisfaction. All three values were below 0.85. These results complement the Fornell-Larcker evidence reported in the manuscript.

**Table S3. Heterotrait-monotrait ratios.**

| **Construct pair 1** | **Construct pair 2** | **HTMT** | **< 0.85** |
| --- | --- | --- | --- |
| Interactive Features | Perceived Learning Outcomes | 0.684 | Pass |
| Interactive Features | Adolescent Satisfaction | 0.542 | Pass |
| Perceived Learning Outcomes | Adolescent Satisfaction | 0.772 | Pass |

**8. Preferred-Platform Robustness Analysis**

Preferred platform was recorded as a single-response demographic variable. A supplementary standardized construct-score model used Bilibili as the reference category and added platform indicators and interaction blocks while leaving the primary latent-variable SEM unchanged.

**Table S4. Preferred-platform control and interaction checks.**

| **Check** | **Estimate / Wald test** | **p** | **Interpretation** |
| --- | --- | --- | --- |
| PLO outcome: IF focal association | 0.601 | < 0.001 | Platform covariates included |
| SAT outcome: IF focal association | 0.116 | 0.003 | Platform covariates + PLO included |
| SAT outcome: PLO focal association | 0.616 | < 0.001 | Platform covariates + IF included |
| IF × platform interaction block (PLO outcome) | χ²(4) = 6.97 | 0.137 | Interaction block not significant |
| IF/PLO × platform interaction block (SAT outcome) | χ²(8) = 5.68 | 0.683 | Combined interaction block not significant |

The Interactive Features × platform interaction block was not significant for the Perceived Learning Outcomes model (Wald χ²(4) = 6.97, p = 0.137), and the combined Interactive Features × platform and Perceived Learning Outcomes × platform interaction block was not significant for the satisfaction model (Wald χ²(8) = 5.68, p = 0.683). These analyses assess coefficient robustness; formal measurement invariance across platforms was not tested.

**9. Alternative Satisfaction Specification**

A second sensitivity analysis separated SAT1–SAT9 as core satisfaction, SAT10–SAT12 as continuance intention, and SAT13–SAT15 as word-of-mouth intention. Standardized regressions controlled for Interactive Features and Perceived Learning Outcomes.

**Table S5. Alternative downstream-intention specification.**

| **Outcome** | **Predictor** | **Std. β** | **p** | **Model R²** |
| --- | --- | --- | --- | --- |
| Continuance intention | Core satisfaction | 0.594 | < 0.001 | 0.466 |
| Continuance intention | Perceived learning outcomes | 0.122 | 0.011 | 0.466 |
| Continuance intention | Interactive features | 0.001 | 0.980 | 0.466 |
| Word-of-mouth intention | Core satisfaction | 0.536 | < 0.001 | 0.469 |
| Word-of-mouth intention | Perceived learning outcomes | 0.175 | < 0.001 | 0.469 |
| Word-of-mouth intention | Interactive features | 0.033 | 0.368 | 0.469 |

Core satisfaction strongly predicted continuance intention (β = 0.594, p < 0.001, R² = 0.466) and word-of-mouth intention (β = 0.536, p < 0.001, R² = 0.469). The primary manuscript retains Adolescent Satisfaction as a broad post-use evaluation, while this alternative specification treats continuance and word-of-mouth as downstream intentions and is reported as a complementary sensitivity analysis.

**10. References Used in Methodological Documentation**

1.McMillan S J, Hwang J S. Measures of perceived interactivity: An exploration of the role of direction of communication, user control, and time in shaping perceptions of interactivity[J]. Journal of advertising, 2002, 31(3): 29-42. doi: 10.1080/00913367.2002.10673674

2. Bandura A. Social foundations of thought and action[J]. Englewood Cliffs, NJ, 1986, 1986(23-28): 2.

3. Zheng Y, Xiao A. A structural equation model of online learning: investigating self-efficacy, informal digital learning, self-regulated learning, and course satisfaction[J]. Frontiers in psychology, 2024, 14: 1276266. doi: 10.3389/fpsyg.2023.1276266

4. Al-Fraihat D, Joy M, Masa'deh R, et al. Evaluating E-learning systems success: An empirical study[J]. Computers in human behavior, 2020, 102: 67-86. doi: 10.1016/j.chb.2019.08.004

5. Leong CM, Goh CF, Ismail F, Tan OK, Ong CH. E-learning satisfaction: Investigating gender differences. International Journal of Electronic Commerce Studies. 2021 Jan 1;12(1):1-28. doi: 10.7903/ijecs.1774
